# Treatment failure and associated factors among people living with HIV on second-line antiretroviral therapy in Zambia

**DOI:** 10.1093/inthealth/ihag024

**Published:** 2026-03-27

**Authors:** Kaziwe Sikambale, Chitalu Chanda, Victor Daka, Eustarckio Kazonga, Steward Mudenda

**Affiliations:** Department of Pharmacy, Mary Begg Hospital, Ndola 10101, Zambia; Department of Public Health, School of Medicine and Health Sciences, University of Lusaka, Lusaka 10101, Zambia; Department of Medicine, Ndola Teaching Hospital, Ndola 10101, Zambia; Public Health of Department, School of Medicine, Copperbelt University, Ndola 10101, Zambia; Department of Public Health, School of Medicine and Health Sciences, University of Lusaka, Lusaka 10101, Zambia; Department of Public Health, School of Medicine and Health Sciences, University of Lusaka, Lusaka 10101, Zambia; Department of Pharmacy, School of Health Sciences, University of Zambia, Lusaka 10101, Zambia; Antimicrobial Resistance Coordinating Committee, Zambia National Public Health Institute, Lusaka 10101, Zambia; Education and Continuous Professional Development Committee, Pharmaceutical Society of Zambia, Lusaka 10101, Zambia

**Keywords:** dolutegravir, HIV treatment failure, risk factors, second-line ART, virological suppression, Zambia

## Abstract

**Background:**

Treatment failure in second-line antiretroviral therapy (ART) among people living with HIV remains a critical concern in Zambia. This study investigated virological failure and factors associated with treatment failure among patients on second-line ART at two tertiary hospitals in Zambia.

**Methods:**

A cross-sectional study was conducted on 257 patient records from 1 January 2022 to 30 April 2025 on second-line ART from two tertiary hospitals. Multivariable logistic regression data analysis was performed using Stata 14.2.

**Results:**

The prevalence of treatment failure was found to be 12.06% (95% CI 8 to 16%). Key predictors of treatment failure included younger age (<15–35 y), male gender (adjusted OR [aOR]–3.05; 95% CI 1.28 to 7.27) and ART initiation at WHO Clinical Stage 2 or above (aOR=1.62; 95% CI 0.68 to 3.82). Additionally, patients on a zidovudine/lamivudine/dolutegravir second-line regimen demonstrated significantly lower odds of failure compared with those on zidovudine/lamivudine/darunavir (aOR=0.34; 95% CI 0.16 to 0.77).

**Conclusions:**

This study found that treatment failure among people living with HIV on second-line ART in Zambia remains a significant concern. To improve treatment outcomes and reduce HIV-associated morbidity and mortality in Zambia, strengthening routine viral load monitoring and adherence support initiatives is essential.

## Introduction

Despite global access to more potent antiretroviral therapy (ART) as part of first-line treatment, treatment failure remains a persistent challenge, particularly in sub-Saharan Africa (SSA), where the HIV burden is highest.^1^ In Zambia, the prevalence of HIV among hospitalized people is persistently high, up to 50%, and approximately 30% of the inpatient admission of people living with HIV (PLHIV) have ART treatment failure.^2^ Furthermore, the postdischarge outcome of mortality rate is as high as 30% among PLHIV with treatment failure compared with those who are HIV negative.^3^ PLHIV with treatment failure are also at an increased risk of hospital readmission postdischarge.^3^

Zambia has made significant strides in HIV/AIDS management, with ART coverage expanding to a large proportion of PLHIV.^4^ The country aligns with global efforts to meet the 95–95–95 targets, yet treatment failure remains a growing concern.^5^ The Zambia Population-Based HIV Impact Assessment showed that viral suppression was at 89.5%.^6^ However, the prevalence of treatment failure among patients on second-line treatment remains unexplored.^6^ The Ndola youth study reported resistance in people failing first-line ART, but only limited data (very small numbers) on those failing second-line who might need third-line.^7^ The patients failing second-line basically are treated with salvage therapy, which has limited options in Zambia.^8^

According to the Zambia Consolidated Guideline, the first-line therapy is predominantly tenofovir/lamivudine/dolutegravir (TLD) for adults and adolescents.^9^ Patients with confirmed virological failure on first-line ART are switched to second-line regimens, which typically consist of a boosted protease inhibitor (PI) lopinavir/ritonavir (LPV-r) or atazanavir/ritonavir (ATV-r) combined with an optimized nucleoside reverse transcriptase inhibitor (NRTI) backbone.^9^ Furthermore, recent updates to the guidelines have included the use of more potent and new molecules such as DTG or darunavir boosted with optimized background as part of second-line ART. Evidence from other SSA countries shows increasing second-line failure, driven by factors such as delayed switching from first-line ART, poor adherence, prior NRTI exposure and PI resistance.^10^

Since the introduction of the more potent second-line options, there has been sparse information regarding treatment failure among PLHIV in Zambia.^11^ Therefore, the current study was conducted to evaluate the treatment failure rate and associated factors among PLHIV on second-line ART at two tertiary hospitals (Ndola Teaching Hospital [NTH] and Kitwe Teaching Hospital [KTH]) in the Copperbelt Province of Zambia. Understanding these factors may be essential for informing future strategies as it may improve treatment outcomes and may mitigate the consequences of virological non-suppression in this vulnerable population.

## Materials and methods

### Study design, setting and population

This study was a retrospective review using routinely collected data from medical records of patients on second-line ART at NTH and KTH. Additionally, we utilized routinely collected data from Zambia’s National Electronic Health Records System (SmartCare) for patients aged ≥15 y receiving ART from 1 January 2022 to 30 May 2025. The study population consisted of PLHIV receiving second-line ART at NTH or KTH. Eligible participants were those on second-line ART for a minimum duration of 6 mo.^6^ Conversely, patients on second-line ART for <6 mo were excluded.

### Study sample

The study sample consisted of participants aged ≥15 y with demographic information who had been switched to a second-line regimen for >6 mo, thus were eligible for inclusion.

The Kish and Leslie formula was used to determine the sample size.^12^


\begin{eqnarray*}
{{\mathbf{n}}} = \,\frac{{{{\mathbf{p}}}\left( {1 - {{\mathbf{p}}}} \right){{{\mathbf{Z}}}}^2{\mathrm{\ }}}}{{{{{\mathbf{d}}}}^2}}
\end{eqnarray*}

Sample size (n) was calculated using the Kish and Leslie sample size formula assuming an 80% proportion of virological suppression, where n=sample size=Z-score (1.96 at 95% confidence level), p=estimated proportion (0.8) and d=margin of errors (5%).^13^ Using these parameters, the minimum required sample size was 266 participants.

Because of the limited number of PLHIV receiving second-line ART at the study sites, consecutive sampling was employed.

### Data collection

Patient information, including sociodemographic characteristics, baseline clinical data and treatment-related variables, was routinely collected from 1 January 2022 to 30 May 2025 by the attending nurse or clinician from medical records and the SmartCare electronic system. We used a structured data abstraction tool adapted from a previous study.^14^ The tool was modified to align with the objectives and specific variables of the present study. Data were entered into a Microsoft Excel 2013 spreadsheet. Key variables included demographic information, ART regimen, viral load (VL) measurements (tested by COBUS), CD4 count (collected using BD FACSPresto) and comorbidities. The choice of second-line ART regimen was determined by the attending clinician according to standard treatment guidelines.

### Data analysis

Data were analyzed using STATA version 14.2. Descriptive statistics were used to summarize participant characteristics and are presented as frequencies and proportions. χ^2^ tests were performed to assess the relationship between treatment failure and selected demographic and clinical factors.

The OR and 95% CI were calculated to assess the strength of the association and only variables with p<0.05 in the logistic regression were considered as statistically significant. Variables that showed significance (p<0.05) in the univariate analysis, as well as those that were clinically important, were included in the multivariable analysis.^15^

The primary outcome was the proportion of second-line HIV treatment failure, defined as virological failure, based on WHO criteria: two successive HIV VL measurements ≥1000 copies/mL taken at least 3 mo apart in an individual who has been on ART for a minimum of 6 mo, and with adherence support provided in the interim.^16^ The secondary outcome was the identification of factors associated with second-line HIV treatment failure.

Multivariable logistic regression analyses were conducted to identify factors associated with treatment failure. Only variables that remained statistically significant at p<0.05 and were considered clinically relevant were retained in the final model.

## Results

### Participant characteristics

A total of 257 participants, 152 (59.1%) from NTH and 105 (40.9%) from KTH, on second-line ART, were included in the analysis. Most participants (71.98%, n=185) were aged >35 y. There were more females (51.47%, n=133) than males (48.53%, n=119) (Table 1).

**Table 1.** Demographic characteristics of participants

Variable	NTH	KTH	Frequency	%
Age group (y)				
15–18	2	1	3	1.17
19–25	14	11	25	9.73
26–35	25	19	44	17.12
>35	111	74	185	71.98
Gender				
Female	80	53	133	51.75
Male	72	52	124	48.25
Residence				
High cost	69	42	111	43.19
Medium	81	60	141	54.86
Other	2	3	5	1.95
Marital status				
Single	46	22	68	26.46
Married	58	62	120	46.69
Widowed	38	7	45	17.51
Divorced	9	4	13	5
Other	1	10	11	4.28
Employment status				
Employed	5	12	17	6.61
Unemployed	146	93	239	93.0
Other	1	0	1	0.39
Total	**152**	**105**	**257**	**100**

KTH: Kitwe Teaching Hospital; NTH: Ndola Teaching Hospital.

### Clinical characteristics

Of 241 patients with documented WHO clinical stage at ART initiation, the majority (74.27%, n=179) commenced treatment at Stage 1. The most frequently used first-line regimen was tenofovir + lamivudine + efavirenz (TLE), which was prescribed to 42.41% (n=109), and the most commonly prescribed second-line regimen was zidovudine/lamivudine/DTG (AZT/3TC/DTG), accounting for 65.37% (n=168) (Table 2).

**Table 2.** Clinical characteristics of participants

Variable	Frequency (n)	%
WHO stage at ART initiation		
Stage 1	179	74.27
Stage 2	3	1.24
Stage 3	59	24.48
Stage 4	0	0
Current comorbidity		
Hypertension	48	18.70
Diabetes	2	0.80
None	207	80.5
ART regimen at initiation		
TLE	109	42.41
TLD	47	18.29
TL-NVP	90	35.02
D4T/3TC/NVP	6	2.33
AZT/3TC/EFV	5	1.95
Current regimen used in second-line ART		
AZT/3TC/DRV-r	81	31.52
AZT/3TC/DTG	168	65.37
AZT/3TC/LPV-r	8	3.11
Total	257	100

3TC: lamivudine; ART: antiretroviral therapy; AZT: zidovudine; D4T/3TC/NVP: Stavudine + Lamivudine + Nevirapine; DRV-r: darunavir–ritonavir; DTG: dolutegravir; LPV-r: lopinavir/ritonavir; TLD: tenofovir/lamivudine/dolutegravir; TLE: tenofovir + lamivudine + efavirenz; TL-NVP: Tenofovir + Lamivudine + Nevirapine.

### Prevalence of treatment failure

The overall prevalence of treatment failure among patients on second-line ART was 12.06% (95% CI 8 to 16%). When stratified by site, the prevalence was 11.84% (95% CI 6 to 17%) at NTH and 12.38% (95% CI 6 to 19%) at KTH.

### Demographic factors associated with virological suppression

Virological suppression among patients on second-line ART at NTH and KTH was assessed in relation to key demographic variables. Significant associations were observed for age group (p=0.016), gender (p=0.013) and employment status (p=0.028) (Table 3).

**Table 3.** Patients’ demographic factors associated with virological suppression at the two tertiary hospitals, Ndola, Zambia

Characteristic	Category	NTH Suppressed, n (%)	NTH Unsuppressed, n (%)	KTH Suppressed, n (%)	KTH Unsuppressed, n (%)	p
Age group (y)						0.016
	15–19	0 (0.0)	2 (100)	1 (100)	0 (0.0)	
	20–25	12 (85.7)	2 (14.3)	11 (100)	0 (0.0)	
	26–35	21 (84.0)	4 (16.0)	15 (78.9)	4 (21.1)	
	>35	100 (90.1)	11 (9.9)	65 (87.7)	9 (12.3)	
Gender	Male	57 (79.2)	15 (20.8)	45 (86.3)	7 (13.7)	0.013
	Female	76 (95.0)	4 (5.0)	47 (88.7)	6 (11.3)	
Marital status	Single	38 (82.6)	8 (17.4)	19 (86.4)	3 (13.6)	0.299
	Married	53 (91.4)	5 (8.6)	55 (90.2)	6 (9.8)	
	Widowed	32 (84.2)	6 (15.8)	5 (71.4)	2 (28.6)	
	Divorced	9 (100)	0 (0.0)	12 (85.7)	2 (14.3)	
	Other	1 (100)	0 (0.0)	0	0	
Residence	High-cost	59 (74.7)	10 (12.6)	38 (90.5)	4 (9.5)	0.695
	Medium	72 (88.9)	9 (11.1)	51 (85)	9 (15)	
	Other	2 (100)	0 (0.0)	3 (100)	0 (0.0)	
Formal employment	Employed	3 (60)	2 (40)	9 (75)	3 (25)	0.028
	Unemployed	130 (88.4)	17 (11.6)	83 (89.2)	10 (10.8)	
Total		133	19	92	13	

KTH: Kitwe Teaching Hospital; NTH: Ndola Teaching Hospital.

Suppression rates were highest among patients aged >35 y at both hospitals, while those aged 15–25 y exhibited the lowest suppression. Female patients demonstrated better suppression outcomes than males at both sites. Interestingly, employed individuals had lower suppression rates than their unemployed counterparts at both facilities (Table 3).

### Clinical factors associated with virological suppression

The relationship between clinical characteristics and virological suppression was evaluated. At NTH, most suppressed patients had initiated ART at WHO Stage 1 (n=131), whereas at KTH, suppression was highest among those who started at Stage 3. The current second-line ART regimen was significantly associated with virological suppression (p=0.018). Patients on the AZT/3TC/DTG regimen exhibited the highest suppression rates at both hospitals (101 at NTH, 52 at KTH), whereas suppression was comparatively lower among those on the AZT/3TC/DRV-r and AZT/3TC/LPV-r regimens (Table 4).

**Table 4.** Clinical factors associated with virological suppression at the two tertiary hospitals

Characteristic	Category	NTH Suppressed, n (%)	NTH Unsuppressed, n (%)	KTH Suppressed, n (%)	KTH Unsuppressed, n (%)	p
WHO stage at initiation of ART						
	1	131 (87.3)	19 (12.7)	24 (82.8)	5 (17.2)	0.488
	2	1 (100)	0 (0.0)	2 (100)	0 (0.0)	
	3	1 (25)	3 (75)	53 (91.4)	5 (8.6)	
current opportunistic infection						
	Currently on treatment for TB	1 (50)	1 (50)	30 (90.9)	3 (9.1)	0.844
	No symptoms	132 (88)	18 (12)	62 (86.1)	10 (13.9)	
Current comorbidities	Hypertensive	27 (90)	3 (10)	15 (100)	0 (0.0)	0.171
	Diabetes	2 (50)	2 (50)	2 (100)	0 (0.0)	
	None	105 (89.0)	13 (11)	75 (85.2)	13 (14.8)	
Current second-line regimen						0.018
	AZT/3TC/DRV-r	30 (81.1)	7 (18.9)	35 (79.5)	9 (20.5)	
	AZT/3TC/DTG	101 (89.4)	12 (10.6)	52 (94.5)	3 (5.5)	
	AZT/3TC/LPV-r	2 (100)	0 (0.0)	6 (100)	0 (0.0)	
Total		133	19	93	12	

3TC: lamivudine; ART: antiretroviral therapy; AZT: zidovudine; DRV-r: darunavir–ritonavir; DTG: dolutegravir; KTH: Kitwe Teaching Hospital; LPV-r: lopinavir/ritonavir; NTH: Ndola Teaching Hospital.

### Factors associated with ART treatment failure

Male patients had more than three times the odds of experiencing treatment failure compared with female patients (adjusted OR [aOR]=3.05; 95% CI 1.28 to 7.27) (Table 5). Compared with patients aged 15–35 y, those aged >35 y were less likely to experience treatment failure, although this association was not statistically significant (aOR=0.47; 95% CI 0.20 to 1.11). Patients who initiated on ART at WHO Clinical Stage 2 or above had slightly higher odds of treatment failure compared with those with Stage 1, but this was not statistically significant (aOR=1.62; 95% CI 0.68 to 3.82). Patients receiving AZT/3TC/DTG were significantly less likely to experience treatment failure compared with those on AZT/3TC/DRV-r (aOR=0.34; 95% CI 0.16 to 0.77) (Table 5).

**Table 5.** Predictors of treatment failure from multivariable analysis

Predictor	cOR (95% CI)	p	aOR (95% CI)	p
Gender				
Female (reference)	1			
Male	2.51 (1.13 to 5.57)	0.02	3.05 (1.28 to 7.27)	0.01
Age group (y)				
15–35	1			
>35	0.57 (0.26 to 1.25)	0.25	0.47 (0.20 to 1.11)	0.08
WHO clinical stage at ART initiation				
Stage 1 (reference)	1			
WHO stage >2	1.26 (0.56 to 2.83)	0.42	1.62 (0.68 to 3.82)	0.27
Second-line ART used				
AZT/3TC/DRV-r (reference)	1			
AZT/3TC/DTG	0.36 (0.16 to 0.78)	0.01	0.34 (0.16 to 0.77)	0.01

3TC: lamivudine; aOR: adjusted OR; ART: antiretroviral treatment; AZT: zidovudine; cOR: crude or unadjusted OR; DRV-r: darunavir–ritonavir; DTG: dolutegravir.

## Discussion

This study assessed the prevalence and predictors of treatment failure among patients on second-line ART at NTH and KTH. The overall treatment failure rate was 12.06%, determined solely through VL monitoring, consistent with WHO recommendations. VL monitoring is the gold-standard approach for assessing treatment efficacy, and its implementation into national health systems is a global health priority if the elimination of HIV as a public health threat is to be achieved by 2030.^17^ The comparable failure rates observed at NTH (11.84%) and KTH (12.38%) suggest similar systemic challenges across facilities. The observed prevalence of treatment failure among patients on second-line therapy is lower than that reported in earlier studies conducted in resource-limited settings.^18^ The observed difference could be attributable to differences in the drugs used in second-line ART, with Zambia now currently using more potent drugs such as DTG and darunavir.

Several clinical and demographic factors were associated with treatment outcomes. Patients initiated on ART at WHO Clinical Stage 2 or above had significantly higher odds of treatment failure compared with those at Stage 1. These findings are consistent with those reported in a study conducted in Uganda that reported that WHO Clinical Stage 2 was significantly associated with increased odds of treatment failure, particularly among individuals aged 26–35 y.^19^ These findings highlight the importance of timely initiation of effective second-line regimens and careful regimen selection to optimize treatment outcomes among patients already diagnosed with HIV. Male patients were three times more likely to experience treatment failure compared with female patients, a finding consistent with a study from eastern Ethiopia, where male patients had greater than fourfold higher odds of treatment failure (aOR=4.49; 95% CI 2.61 to 7.73).^20^ This could be explained by the lack of male-friendly health services and poor health-seeking behaviors among men, which together contribute to reduced adherence and retention in care.

Participants aged 15–35 y were significantly more likely to fail therapy than those aged >35 y; although our study did not find age to be a significant predictor, literature exists showing that adolescents and youths living with HIV have worse outcomes at all stages of the care cascade compared with adults.^21^ Several factors may contribute to this finding, including psychosocial and behavioral challenges that are more pronounced in this age group.^22^ However, given that only a few participants in this category were included across both facilities, this finding should be interpreted with caution due to the small sample size. In particular, adolescents’ daily routines and emotional instability have been shown to act as barriers to consistent medication adherence.^23^ Furthermore, adherence is often negatively impacted by the fear of accidental disclosure of their HIV status, which may lead to skipped doses or a reluctance to engage fully with care services.^24^ These findings underscore the need for youth-friendly HIV care models that address the unique social and emotional needs of adolescents to improve treatment outcomes.^25^

Although hypertension, diabetes and TB coinfection were not statistically associated with treatment failure in this cohort, prior studies have shown that these comorbidities may contribute to poorer ART outcomes due to drug-drug interactions, increased pill burden and immune suppression.^26^ However, these findings are in line with those of Mehari and colleagues in their retrospective observational study on DTG-based regimens in Ethiopia, which found that the presence of hypertension and diabetes did not significantly impact virological suppression outcomes before and after switching to DTG-based therapy.^27^ This suggests that common non-communicable comorbidities may not independently influence ART effectiveness in resource-limited settings.

Similarly, while marital status, residence and employment did not show statistically significant associations, employed individuals had more than twice the odds of experiencing treatment failure compared with unemployed individuals, and widowed and divorced individuals showed higher rates of virological non-suppression, suggesting potential social or economic barriers affecting adherence. These findings contrast with results from a systematic review and meta-analysis conducted by Orievulu et al. in Ethiopia, which found that place of residence significantly affected adherence to ART. Specifically, patients living in urban areas were 2.07 times more likely to have good adherence compared with those in rural settings.^28^

The type of second-line ART regimen significantly influenced treatment outcomes. Patients receiving AZT/3TC/DTG had significantly lower odds of treatment failure (aOR=0.34; p=0.01) compared with those on AZT/3TC/DRV-r, underscoring the benefits of DTG-based regimens.^29^ These findings are consistent with results from the NADIA trial, which demonstrated that recycling DTG remains superior to switching to a PI-based regimen.^30^

This study identifies some key risk factors, such as age, gender, WHO clinical stage and specific ART regimens, which were significantly associated with treatment failure; it also identified vulnerable groups such as adolescents and males. Therefore, local management of these clients can be tailored to specific variables to improve their outcomes.

We are aware that our study has some limitations. Adherence data were not available, despite adherence being a critical determinant of treatment outcomes. Further investigation of patient-related factors influencing adherence is therefore an important area for future research to better elucidate the causes of treatment failure. The current study relied on secondary data from medical records, which may be incomplete or prone to documentation bias. The study was also limited to two hospitals, which may affect the generalizability of its findings to other settings or regions.

### Conclusions

This study found a treatment failure of 12.06% among individuals on second-line ART living with HIV at NTH and KTH in the Copperbelt Province of Zambia. While the 12.06% treatment failure rate appears modest, it is notable that this level falls short of the UNAIDS third 95 target, which aims for 95% viral suppression among those on ART by 2030. Additionally, the study identified age, gender and WHO clinical stage as demographic and clinical factors significantly associated with treatment failure, while being on an AZT/3TC/DTG second-line regimen was associated with significantly lower odds of treatment failure. In this cohort, suppression rates across subgroups ranged from 87% to 90%, indicating progress, but also persistent gaps. Given the increasing burden of patients transitioning to second-line therapy in resource-limited settings and the limited availability of third-line regimens, there is a critical need to improve treatment durability.

The findings of the current study support the need for differentiated care strategies targeting high-risk groups, particularly adolescents, males and patients with advanced disease at ART initiation. Expanding access to DTG-based regimens, enhancing adherence counseling and strengthening routine VL monitoring are essential for improving outcomes and moving closer to achieving global HIV control targets.

## Data Availability

The data presented in this study, including the tools employed, are available upon reasonable request from the corresponding author.
